# Discussion table on psychology

**DOI:** 10.1093/eurpub/ckac129.233

**Published:** 2022-10-25

**Authors:** J Niess

**Affiliations:** 1 Human-Computer-Interaction, University of St. Gallen, St. Gallen, Switzerland; 2 Leibniz ScienceCampus Digital Public Health, Bremen, Germany

## Abstract

Jasmin Niess has a background in psychology and focuses on human-computer interaction. She will support the workshop participants during the world coffee at the discussion table for psychology.

